# Treatment outcomes of extended-field radiation therapy and the effect of concurrent chemotherapy on uterine cervical cancer with para-aortic lymph node metastasis

**DOI:** 10.1186/s13014-014-0320-5

**Published:** 2015-01-13

**Authors:** Hong In Yoon, Jihye Cha, Ki Chang Keum, Ha Yoon Lee, Eun Ji Nam, Sang Wun Kim, Sunghoon Kim, Young Tae Kim, Gwi Eon Kim, Yong Bae Kim

**Affiliations:** Department of Radiation Oncology, Yonsei Cancer Center, Yonsei University College of Medicine, 50-1 Yonsei-ro, Seodaemun-gu, Seoul, 120-752 Korea; Department of Radiation Oncology, Yonsei University Wonju College of Medicine, Wonju, Korea; Division of Gynecologic Oncology, Department of Obstetrics and Gynecology, Yonsei University College of Medicine, Seoul, Korea; Department of Radiation Oncology, Jeju National University School of Medicine, Jeju, Korea; Department of Pharmacology, Brain Korea 21 plus Project for Medical Science, Yonsei University College of Medicine, Seoul, Korea; Yonsei Song-Dang Institute for Cancer Research, Yonsei University College of Medicine, Seoul, Korea

**Keywords:** Cervical cancer, Treatment outcome, Chemotherapy, Para-aortic lymph node, Extended-field radiation therapy

## Abstract

**Purpose:**

To review the clinical outcomes of extended-field radiation therapy (EFRT) and to analyze prognostic factors significant for survival in patients receiving EFRT for uterine cervical carcinoma with para-aortic node (PAN) metastasis.

**Patients and methods:**

We retrospectively reviewed 90 patients with stage IB-IVA cervical cancer and PAN metastasis between 1987 and 2012. Median age was 50 (range, 24–77). Patients received median 70.2 Gy (range, 56–93) to point A and median 50.4 Gy (range, 45–60.4) to PAN over median 69 elapsed days (range, 43–182). Forty-six patients (51.1%) received concurrent chemotherapy. Survival was calculated using the Kaplan–Meier method. We analyzed prognostic factors for overall actuarial survival (OS) and progression-free survival (PFS) using a Cox regression method.

**Results:**

The median follow-up period for surviving patients was 55 months (range, 3–252). Seventy patients (77.8%) had complete remission. Forty-six patients experienced treatment failure as follows: 11 patients (12.2%) as local recurrence, 19 (21%) as regional recurrence and 33 (36.7%) as distant metastasis. The 5-yr OS and PFS were 62.6% and 43.9%, respectively. Treatment response was the only statistically independent prognostic factors for OS (p= 0.04) and PFS (p< 0.001) on multivariate analysis. Grade 3 or 4 hematologic gastrointestinal and urogenital toxicities were observed in about 10% of patients.

**Conclusions:**

Our institutional experiences showed that EFRT was an effective treatment for cervical cancer patients with PAN metastasis. The addition of chemotherapy to EFRT seems to have uncertain survival benefit with higher hematologic toxicity.

**Electronic supplementary material:**

The online version of this article (doi:10.1186/s13014-014-0320-5) contains supplementary material, which is available to authorized users.

## Introduction

Para-aortic node (PAN) metastasis is an important prognostic factor, although the International Federation of Gynecology and Obstetrics (FIGO) staging system of uterine cervical cancer does not allow for the involvement of nodes [1]. According to the Gynecologic Oncology Group, biopsy-confirmed PAN involvement was found in 5% of Stage IB patients, 17% of Stage IIB patients, and 25% of Stage IIIB patients; poor survival rates of these patients were reported [2].

Extended-field radiation therapy (EFRT) is conventionally indicated for cervical cancer patients with grossly detected common iliac or PAN metastasis. In several previous studies on EFRT, a 5-year overall survival rate of 29 – 32% was reported [3-5]. In some prospective and retrospective trials with EFRT plus concurrent chemotherapy, severe toxicity of EFRT plus concurrent chemotherapy, including gastrointestinal or hematologic toxicity, was observed despite a favorable survival rate [6-8]. Therefore, some investigators have tried to reduce the toxicity of EFRT plus concurrent chemotherapy using intensity-modulated radiotherapy or low dose chemotherapy [9,10]. However, despite a large number of small population studies, the effect of EFRT with or without concurrent chemotherapy has still not been elucidated.

The purpose of this study was to investigate the clinical outcomes, including toxicity, treatment response, patterns of failure, and survival, as well as to analyze prognostic factors significant for survival, in patients receiving EFRT for uterine cervical carcinoma with PAN metastasis.

## Methods and materials

### Patient selection and characteristics

This retrospective study received approval from the internal review boards of the participating institution (IRB No. 4-2014-0162). Between April 1987 and December 2012, 123 patients with cervical cancer and PAN metastasis were treated with EFRT at our institution. Among them, 33 patients were excluded if any of the following conditions were met: (1) stage IVB, (2) distant nodal metastasis in inguinal, mediastinal or supraclavicular lymphatics, (3) salvage, palliative, or postoperative RT, (4) induction or post-RT chemotherapy, (5) incomplete RT due to patient refusal. Consequently, 90 patients were included in this retrospective study. Clinical staging was based on the FIGO stage classifications updated in 2009 [11]. The procedure for staging included a detailed history and a physical examination, common laboratory tests, standard chest radiographs, intravenous pyelograms, barium enemas, X-ray examination of the lungs and skeleton, cystoscopies, and sigmoidoscopies. All patients underwent computed tomography (CT) (57.8%) or magnetic resonance imaging (MRI) (63.3%) scans to evaluate pelvic or para-aortic lymph node involvement. Positron emission tomography (PET) or PET-CT scans were performed in 21 patients (23.3%). In the image interpretation of CT or MRI, the principal criterion for metastatic node involvement was the axial diameter of the lymph node. The presence of lymph nodes larger than 1 cm in the short-axis dimension was considered to indicate metastatic node involvement. Additionally, we regarded central necrosis as a significant criterion for metastatic disease within the lymph node [12]. In the image interpretation of PET or PET-CT, a malignant lymphadenopathy was defined as follows: 1) fluorodeoxyglucose (FDG) accumulation greater than liver accumulation or similar to brain cortex accumulation, or 2) standardized uptake value of a lesion that corresponded to CT and did not decrease on the delayed PET image compared with the initial PET image [13]. Para-aortic lymph nodes were surgically assessed in 7 patients (7.8%). Histologic classification was based on the World Health Organization classifications (Geneva, Switzerland).

### Radiotherapy

All patients received a combination of external EFRT and high-dose-rate intracavitary brachytherapy (HDR-ICR). We used the box technique with parallel opposing fields for 87 patients or the two-field technique with antero-posterior fields for 3 patients. For para-aortic irradiation, we defined the T11-T12 or T12-L1 interspace covering the entire PAN as the superior border, 2 cm from the front of the vertebral body or enlarged lymph nodes as the anterior border, and the midline of the vertebral body as the posterior border, respectively. For whole pelvic irradiation, we defined the inferior border of the obturator foramen (if distal vaginal was not involved) or 2 cm below the lowest extent of the primary tumor (if there was distal vaginal invasion) as the inferior border, and 1.5 cm to 2 cm from the true bony pelvis as the lateral border in AP-PA fields. External EFRT was delivered using a 10-MV linear accelerator with a dose of 1.8-2 Gy per fraction, 5 times per week. Midline shielding with a 4 cm-width was performed after the delivery of 26.0 to 45 Gy based on treatment response. This was followed by HDR-ICR using a remote afterloading system with a Ralstron 303 Co-60 source (Shimadzu, Kyoto, Japan) from 1979 to 1997, or a Gamma-Med II Ir-192 source (Sauerwein, Haan, Germany) from 1989 to 2006, or Multisource® Ir-192 source (Eckert & Ziegler BEBIG, Berlin, Germany) from 2007 onward. The total dose of HDR-ICR was 20–48 Gy, with 3 or 5 Gy per fraction, which was prescribed to point A. Overall, a 3 Gy per fraction with a median fraction number of 10 (range, 8–16) was prescribed for 43 patients and a 5 Gy per fraction with a median fraction number of 6 (range, 4–8) was administered to the others. After the completion of HDR-ICR, patients were administered a second course of external EFRT with midline shielding to a total external beam dose of 45 to 54 Gy. For patients with persistent residual disease, which was identified on pelvic examination and imaging studies performed at 1 month after planned EFRT and HDR-ICR, boost irradiation to the parametrium, pelvic wall, and involved node was performed. The median dose of boost irradiation was 9 Gy (range, 5.4-16 Gy).

### Chemotherapy

Platinum single-agent or platinum-based doublet regimens were used. For concurrent chemoradiation (CCRT), protocols included two treatment schemes. One scheme was composed of three chemotherapy cycles administered at the beginning of the first, fourth, and seventh weeks of RT [14], and the regimen consisted of cisplatin (70 mg/m^2^) or carboplatin (area under the curve (AUC), 4) followed by five consecutive daily infusions of 5-FU (1000 mg/m^2^/day). Weekly administration of cisplatin (40 mg/m^2^) or carboplatin (AUC, 2) during RT has been performed since the publication of randomized trials [15].

### Follow-up

During treatment, adverse effects and performance levels were monitored weekly. After completion of treatment, all patients were evaluated at 1 month and every 3 months for the first 2 years, and every 6 months thereafter. Acute treatment-related hematologic toxicities were defined according to the National Cancer Institute Common Terminology Criteria for Adverse Events, version 4.0. Acute toxicities were evaluated from the start of treatment to 3 months following the completion of treatment. Depending on the severity and duration of toxicity, treatment was interrupted until the patient recovered. Late treatment-related toxicities were assessed using the Late Radiation Morbidity Scoring Scheme of the Radiation Therapy Oncology Group and the European Organization for Research and Treatment of Cancer. Late radiation toxicities developing later than 6 months after the completion of treatment were grouped into rectal, bladder, small bowel, and other complications.

We defined a complete remission (CR) as 100% decrease of gross tumor on clinical evaluation or radiologic images. Partial response (PR) and progressive disease (PD) were defined as ≥ 50% decrease and > 25% increase of primary gross tumor, respectively. Anything else was categorized as stable disease. We evaluated treatment responses by performing history taking, physical and pelvic examinations, and imaging studies, such as MRI or CT scan, at 3 months after completion of all treatments. The follow-up imaging studies were performed routinely at 1, 3, 6, 12, 18, and 24 months after treatment completion, and once a year thereafter. We defined local recurrence as any relapse or persistent disease at the cervix, vagina, parametrium, or pelvic wall. Regional recurrence was defined as node relapse within the RT field. Although PAN relapse is considered as a distant metastasis in the TNM staging system, PAN relapse was classified as a regional recurrence in this study. We defined distant metastasis as relapse outside the RT field.

### Statistical analysis

Total dose to point A (Gy) was calculated by combining the dose of external beam irradiation before midline shielding and the total dose to point A in HDR-ICR. Overall actuarial survival (OS) and progression-free survival (PFS) were calculated using the Kaplan–Meier method and differences in survival rates were compared by the log-rank test. The OS time was calculated from the date of RT start to the date of death or last follow-up. The PFS time was calculated from the date of RT start to the date of disease progression, relapse, initiation of new unplanned anticancer therapy, disease-related death, or last follow-up. We analyzed prognostic factors for OS and PFS using a Cox regression method. Significant variables on univariate analysis were utilized for multivariate analysis to establish independent prognostic factors for OS and PFS. Differences in nominal variables were compared using Pearson’s χ^2^ test or Fisher's exact test. We analyzed difference in continuous variables using the Mann–Whitney *U* test. We considered statistical significance as p value ≤ 0.05.

## Results

### Patient and treatment characteristics

All patient and treatment characteristics are listed in Tables 1 and 2, respectively. Patients received a median of 70.2 Gy to point A and a median of 50.4 Gy to PAN over a median of 69 elapsed days. Forty-six patients (51.1%) received concurrent chemotherapy.

**Table 1:** **Patient characteristics**

**Characteristics**	**Total patients (n = 90)**	
	**n**	**(%)**
Age		
Median	50	
Range	(24–77)	
ECOG performance		
0	55	61.1
1	33	36.7
2	2	2.2
Pathologic findings		
Squamous cell carcinoma (SCC)	84	93.3
Large cell keratinizing	18	20
Large cell non-keratinizing	47	52.2
Large cell, not specified	3	3.3
SCC, not specified	14	15.6
Small cell	2	2.2
Others (adenocarcinoma, adenosquamous carcinoma)	6	6.7
Tumor shape		
Exophytic	38	42.2
Infiltrative	52	57.8
Parametrial involvement		
No	10	11.1
Yes	80	88.9
Unilateral	42	46.7
Bilateral	38	42.2
Endocervical extension		
No	6	6.7
Yes	37	41.1
Not confirmed	47	52.2
Primary tumor size (cm)		
Median	5	
Range	(2–10)	
Pelvic LN involvement	73	81.1
Paraaortic LN involvement	90	100
Hydronephrosis	18	20
FIGO stage		
IB	10	11.1
IIB	39	43.3
IIIA	2	2.2
IIIB	33	36.7
IVA	6	6.7
Treatment period		
Before 2000	52	57.8
2000-present	38	42.2

*Abbreviation:* *ECOG* Eastern Cooperative Oncology Group performance.

**Table 2:** **Treatment characteristics**

**Characteristics**	**n**	**(%)**
Radiotherapy field		
Whole pelvis and lower para-aortic lymphatics	4	4.4
Whole pelvis and entire para-aortic lymphatics	86	95.6
Chemotherapy		
None	44	48.9
Concurrent chemotherapy	46	51.1
Weekly cisplatin or carboplatin	24	26.6
Cisplatin or carboplatin followed by 5-FU	22	24.5
Total dose to point A (Gy)		
Median	70.2	
Range	(56–93)	
Interquartile range (75th and 25th percentiles)	14.4 (80.4,66)	
Total dose to para-aortic lymphatics		
Median	50.4	
Range	(45–60.4)	
Interquartile range (75th and 25th percentiles)	3.6 (54, 50.4)	
Radiotherapy duration (days)		
Median	69	
Range	(43–182)	
Interquartile range (75th and 25th percentiles)	20 (80, 60)	

### Treatment-related toxicities

Fourteen patients (15.6%) exhibited grade 3 or 4 acute leukopenia, 12 (13.3%) exhibited grade 3 or 4 acute anemia, and 12 (13.3%) exhibited grade 3 or 4 acute thrombocytopenia. Grade 3 late gastrointestinal and urogenital toxicities were observed in 8 (8.9%) and 3 patients (3.3%), respectively. There was no grade 5 acute or late treatment-related toxicity.

### Treatment responses, patterns of failure, and survival analyses

Seventy patients (77.8%) had CR and 20 patients (22.2%) had PR. No SD or persistent disease was observed in any patient. For PAN metastasis only, CR was observed in 75 patients (83.3%) and PR in 15 patients (16.7%). Forty-six patients experienced treatment failure (Table 3).

**Table 3:** **Patterns of failure**

**Patterns of failure**	**n**	**(%)**
Local recurrence	11	12.2
Cervix	7	7.8
Vagina	3	3.3
Parametrium/pelvic wall	2	2.2
Regional recurrence	19	21
Pelvic lymph node	10	11.1
Paraaortic lymph node	13	14.4
Distant metastasis	33	36.7
Outfield nodal failure	21	23.3
Bone	4	4.4
Viscera	12	13.3
Liver	4	4.4
Lung	7	7.8
Spleen	1	1.1
Ureter	1	1.1
Carcinomatosis	5	5.6

**Figure 1:**
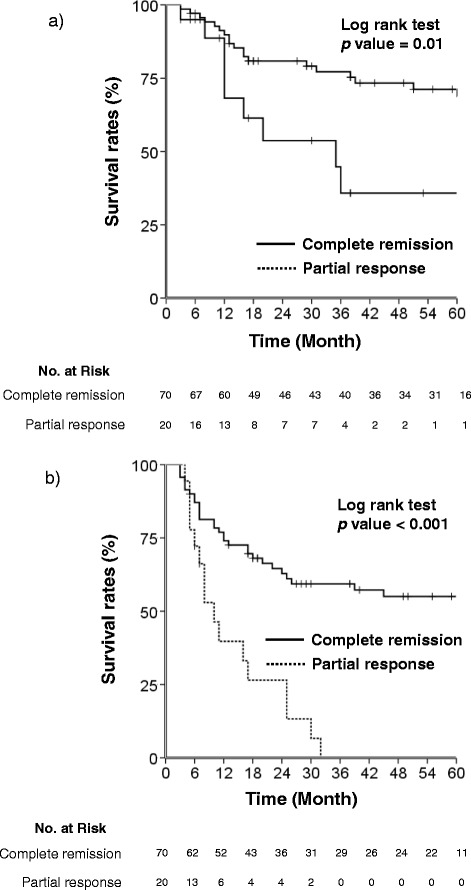
**Kaplan–Meier curves of (a) 5-year overall survival rate (log rank test p = 0.01) and (b) progression-free survival rate (log rank test p < 0.001) according to the treatment response.**

The median follow-up period for surviving patients was 55 months (range, 3–252). The 5-year OS and PFS for all patients were 62.6% and 43.9%, respectively. In univariate analysis, ECOG PS (0 vs. 1 or 2, 5-yr OS 72.3% vs. 44.5%, p = 0.04) and treatment response (CR vs. PR, 5-yr OS 68.8% vs. 35.8%, p = 0.01) showed a significant effect on OS. Using multivariate analysis, treatment response was the only statistically significant factor for OS (p = 0.04, hazard ratio (HR) 0.43, 95% confidence interval (95% CI) 0.19-0.98, Table 4 and Figure 1a). PFS was significantly associated with ECOG PS (0 vs. 1 or 2, 5-yr PFS 54.4% vs. 23.9%, p = 0.02) and treatment response (CR vs. PR, 5-yr PFS 55.0% vs. 0%, p < 0.001) in the univariate analysis, and tumor shape showed significant trends for worse PFS. In the multivariate analysis, only treatment response had a significant influence on PFS (p < 0.001, HR 0.3, 95% CI 0.16-0.58, Table 4 and Figure 1b). Treatment response (CR vs. PR) in PAN metastasis only was also associated significantly with OS (HR 0.25, 95% CI 0.11-0.58, p = 0.001) and PFS (HR 0.27, 95% CI 0.14-0.54, p < 0.001).

**Table 4:** **Prognostic factors on overall survival and progression-free survival by Cox proportional-hazards model**

	**Overall survival**				**Progression-free survival**			
	**Univariate analysis**		**Multivariate analysis**		**Univariate analysis**		**Multivariate analysis**	
**Variables**	**HR (95% CI)**	**p value**	**HR (95% CI)**	**p value**	**HR (95% CI)**	**p value**	**HR (95% CI)**	**p value**
Age (<50 vs. ≥50)	1.21 (0.59-2.51)	0.61			1.24 (0.69-2.21)	0.47		
ECOG PS (0 vs. 1 or 2)	0.45 (0.22-0.95)	0.04	0.53 (0.25-1.12)	0.1	0.48 (0.27-0.87)	0.02	0.57 (0.31-1.03)	0.06
Tumor shape (exophytic vs. infiltrative)	1.75 (0.85-3.64)	0.13			1.75 (0.98-3.14)	0.06	1.67 (0.93-2.99)	0.09
Tumor size (<5 vs. ≥5 cm)	0.67 (0.3-1.47)	0.31			0.72 (0.39-1.33)	0.29		
Parametrial involvement (no vs. yes)	0.4 (0.1-1.7)	0.22			0.5 (0.18-1.39)	0.18		
Endocervical extension (others vs. yes)	1.85 (0.82-4.17)	0.14			1.47 (0.8-2.7)	0.22		
Pelvic nodal involvement (no vs. yes)	0.78 (0.32-1.91)	0.58			0.71 (0.34-1.47)	0.35		
Hydronephrosis (no vs. yes)	1.21 (0.42-3.5)	0.72			0.82 (0.4-1.65)	0.57		
Stage (IB vs. IIB vs. IIIA-IVA)	Referent 0.38 (0.09-1.7)	0.45			Referent 0.45 (0.15-1.3)	0.32		
	0.9 (0.42-1.92)				0.81 (0.44-1.49)			
Radiotherapy duration (<10 vs. ≥10 weeks)	0.59 (0.28-1.23)	0.16			0.62 (0.35-1.12)	0.11		
Doses to point A (<75 vs. ≥75 Gy)	0.87 (0.42-1.8)	0.71			0.64 (0.36-1.15)	0.14		
Doses to PAN (<50.4 vs. ≥50.4 Gy)	1.09 (0.46-2.55)	0.85			0.75 (0.35-1.6)	0.45		
Treatment period (<2000 vs. ≥2000)	1.25 (0.59-2.65)	0.56			0.98 (0.54-1.76)	0.95		
Treatment response (CR vs. PR)	0.36 (0.16-0.81)	0.01	0.43 (0.19-0.98)	0.04	0.27 (0.14-0.51)	<0.001	0.3 (0.16-0.58)	<0.001
Concurrent chemotherapy (no vs. yes)	1.23 (0.59-2.54)	0.59			0.88 (0.49-1.57)	0.65		

*Abbreviation:* *ECOG PS* Eastern Cooperative Oncology Group performance status, *CR* complete remission, *PR* partial response, *HR* hazard ratio, *CI* confidence interval.

### Does the concurrent chemotherapy provide survival benefits?

**Figure 2:**
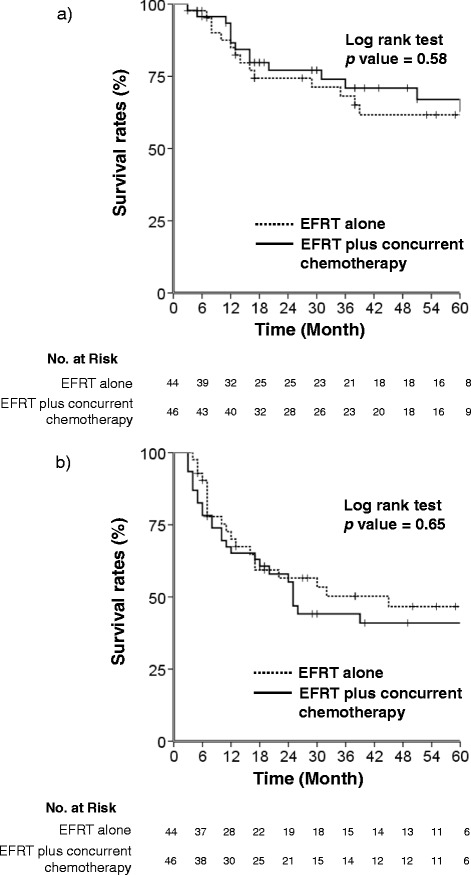
**Kaplan–Meier curves of (a) 5-year overall survival rate (log rank test p = 0.58) and (b) progression-free survival rate (log rank test p = 0.65) according to the addition of concurrent chemotherapy to EFRT.**

The addition of concurrent chemotherapy was not related to OS or PFS in our univariate analysis (Table 4 and Figure 2). Comparing toxicities, we observed severe acute hematologic toxicity (grade 3 to 4) significantly more often in patients receiving EFRT plus concurrent chemotherapy then in those receiving EFRT alone (Table 5). Although severe acute toxicity upon the addition of chemotherapy did not generate a statistical difference in treatment duration (p = 0.18, median 67 vs. 71 days), patients showing severe acute hematologic toxicity in the EFRT plus chemotherapy group showed longer treatment durations (median 81 days, 43–182) than the others (median 64 days, 47–129, p < 0.001). Radiation-induced proctitis, cystitis, enteritis, and vesicovaginal fistula were observed as severe late toxicities. The interval between the start of treatment and the appearance of toxicities was a median of 15 months; the interval in the EFRT plus chemotherapy group was shorter than that for the EFRT alone group (median 13 vs. 27 months, respectively). There was no significant difference in treatment response (CR and PR rates, 75% and 25% in EFRT alone vs. 80.4% and 19.6% in EFRT plus concurrent chemotherapy, p = 0.42) or patterns of failure according to addition of chemotherapy (Additional file 1: Table S1).

**Table 5:** **Treatment-related toxicity according to concurrent chemotherapy in extended-field radiation therapy**

	**Grade 3-4**		**p value**
**Toxicity**	**None (n=44)**	**Concurrent CTx (n=46)**	
Acute			
Leukopenia	0 (0.0)	14 (30.4)	<0.001
Anemia	0 (0.0)	12 (26.1)	<0.001
Thrombocytopenia	0 (0.0)	12 (26.1)	<0.001
Late			
Rectal	4 (9.1)	4 (8.7)	1
Bladder	3 (6.8)	0 (0.0)	0.11
Small bowel	0 (0.0)	1 (2.2)	1
Vagina vault necrosis	0 (0.0)	0 (0.0)	-

*Abbreviation:* *CTx* concurrent chemotherapy.

## Discussion

This study showed that EFRT was an efficient treatment for uterine cervical cancer with involved PAN, with a 5-year OS of 62.6% and PFS of 43.9%. Treatment response was an independent prognostic factor for survival. The addition of concurrent chemotherapy to EFRT did not affect treatment response, patterns of failure, OS, or PFS, but rather, was associated with severe treatment-related toxicity.

Although patients who underwent EFRT alone were irradiated with a median of 54 Gy without a radio-sensitizing chemotherapeutic agent, treatment response rates were not different from patients receiving a median of 50.4 Gy in EFRT plus concurrent chemotherapy. A previous study demonstrated that concurrent chemotherapy did not affect overall survival in isolated PAN recurred cervical cancer patients receiving a median of 50.8 Gy of RT to PAN (p = 0.69) [16]. Therein, a total dose of ≥ 51 Gy tended to show higher survival than that of <51 Gy (p = 0.07). Based on that previous study, we also considered that 54 Gy of EFRT alone would be sufficient to control PAN metastasis without radio-sensitizing chemotherapy. Nevertheless, further study is necessary.

For patients initially diagnosed with PAN metastasis, the efficacy of concurrent chemotherapy seems to be uncertain. Since Stryker et al. reported that cisplatin-based chemotherapy may be beneficial [3], many studies have continued to report favorable outcomes, despite more acute severe hematologic and gastrointestinal toxicity [7-9,17,18]. In reviewing our results, we did not observe a significant survival benefit for the addition of concurrent chemotherapy to EFRT, although the group that received EFRT alone included more patients with disease of advanced stage (p < 0.001, Additional file 1: Table S2). This implies that survival benefit of EFRT plus concurrent chemotherapy is insignificant for uterine cervical cancer with PAN metastasis, suggesting that more effective chemotherapeutic regimen and scheme would be required.

However, along with no difference in PFS according to the addition of chemotherapy, the OS rates between EFRT alone and EFRT plus concurrent chemotherapy also showed no statistically significant difference. Due to the different periods of treatment according to the addition of chemotherapy (Additional file 1: Table S2), we questioned whether differences in therapeutic standards, such as salvage treatment, induced by the treatment year could affect survival. Advances in RT [19-21] and chemotherapy [22,23] over the years have increased the success rates of salvage treatments [24,25]. In this study, patients treated with EFRT plus concurrent chemotherapy received more salvage treatments (80.0% vs. 52.4%, p = 0.047), especially salvage chemotherapy (64% vs. 23.8%, p = 0.006), after relapse (Additional file 1: Table S3). In the survival analysis of relapsed patients only, patients receiving salvage treatment showed significantly improved OS, compared to those receiving only conservative care (median OS 51 vs. 13 months, p < 0.001, Additional file 2: Figure S1). Therefore, it is possible that different treatment years may result in survival differences according to the addition of chemotherapy in this study. The effect of additional chemotherapy to EFRT in uterine cervical cancer with metastatic PAN warrants investigation via a randomized clinical trial. However, since enrolling participants in an EFRT alone group in a prospective trial would be difficult, we are limited to indirect investigations through retrospective trials.

Special considerations are needed to understand our findings because of several drawbacks. Since this study is a retrospective review covering a long period (over 20 years), heterogeneity of patient characteristics might have confused diagnosis, treatment, follow-up, and our results, including treatment response and toxicity. Viewed in this light, the most important consideration is the accuracy of the PAN metastasis diagnosis. PAN metastasis was pathologically confirmed in a small number of patients of this study. Most cases depended on several imaging studies, including CT, MRI, PET, and PET-CT, due to the development of imaging tools over a long time [26-29]. However, as all patients underwent CT or MRI scans in this study, we expect that there would not be many differences in initial staging of lymph node involvement, despite a long treatment period. As well, the long period could have affected whether or not concurrent chemotherapy was undertaken: the benefits of cisplatin-based concurrent chemoradiotherapy were reported in 1999 [30]. Accordingly, after 2000, the EFRT alone group in this study comprised only 9.1% of patients, and most patients were treated with EFRT plus concurrent chemotherapy. Accordingly, we evaluated treatment period as a prognostic factor in the univariate and multivariate analyses of survival rates. However, treatment period did not affect OS and PFS significantly. Chemotherapeutic regimens and schemes were also heterogeneous, which might have influenced treatment outcomes. On prognostic factors analysis using a Cox regression method confined to patients receiving concurrent chemotherapy, difference in chemotherapy scheme was not a significant prognostic factor for OS and PFS (data not shown). Since all patients who underwent EFRT plus concurrent chemotherapy in this study received platinum-based regimens [30,31] and since the addition of chemotherapy and difference in chemotherapy scheme did not affect survival, we do not consider the heterogeneity of chemotherapeutic regimens and schemes to have affected OS and PFS.

In conclusion, we suggest that EFRT with or without concurrent chemotherapy can be an effective treatment for cervical cancer patients with para-aortic node metastasis. Treatment response was a significant prognostic factor for OS and PFS, respectively. Our findings showed the controversial effects of the addition of concurrent chemotherapy to EFRT. Although they should be interpreted cautiously, due to the heterogeneity in this study, our findings deserve consideration, since performing a randomized clinical trial would be impractical.

## Additional files

Additional file 1: Table S1. Patterns of failure according to the addition of chemotherapy. **Table S2.** Patient and treatment characteristics according to the addition of chemotherapy. **Table S3.** Salvage treatments after first recurrence according to treatment modality in relapsed patients. Additional file 2: Figure S1. Kaplan–Meier curve depicts that patients receiving salvage treatment showed significantly improved overall survival rates compared to those receiving only conservative care after relapse (median OS 51 vs. 13 months, p < 0.001).
