# Supplementary material for: Treatment outcomes of extended-field radiation therapy and the effect of concurrent chemotherapy on uterine cervical cancer with para-aortic lymph node metastasis
Source: Radiat Oncol. 2015 Jan 13;10:18. doi: 10.1186/s13014-014-0320-5 (PMC4311470; doi:10.1186/s13014-014-0320-5)
Supplement: Additional file 1: Table S1. — Patterns of failure according to the addition of chemotherapy. Table S2. Patient and treatment characteristics according to the addition of chemotherapy. Table S3. Salvage treatments after first recurrence according to treatment modality in relapsed patients. [file 13014_2014_320_MOESM1_ESM.docx]

Table S1 Patterns of failure according to the addition of chemotherapy

| Failures | EFRT alone (n=44) | | EFRT plus CTx (n=46) | | *p* value |
| --- | --- | --- | --- | --- | --- |
|  | n | (%) | n | (%) |  |
| Local recurrence | 7 | 16 | 4 | 8.7 | 0.3 |
| Cervix | 4 | 9 | 3 | 6.5 | 0.71 |
| Vagina | 3 | 6.8 | 0 | 0.0 | 0.11 |
| Parametrium/pelvic wall | 1 | 2.2 | 1 | 2.2 | 1 |
| Regional recurrence | 8 | 18.2 | 11 | 23.9 | 0.51 |
| Pelvic lymph node | 4 | 9.1 | 6 | 13.0 | 0.74 |
| Paraaortic lymph node | 6 | 13.6 | 7 | 15.2 | 0.83 |
| Distant metastasis | 16 | 36 | 17 | 37 | 0.95 |
| Outfield nodal failure | 10 | 23 | 11 | 23.9 | 0.89 |
| Bone | 1 | 2.3 | 3 | 6.5 | 0.62 |
| Viscera | 6 | 13.6 | 6 | 13.0 | 0.93 |
| Liver | 2 | 4.5 | 2 | 4.3 | 1 |
| Lung | 4 | 9.1 | 3 | 6.5 | 0.71 |
| Spleen | 0 | 0 | 1 | 2.2 | 1 |
| Ureter | 0 | 0 | 1 | 2.2 | 1 |
| Carcinomatosis | 3 | 6.8 | 2 | 4.3 | 0.67 |

Abbreviation: CTx, chemotherapy

Table S2 Patient and treatment characteristics according to the addition of chemotherapy

| Characteristics | EFRT alone (n= 44) | | EFRT plus CTx (n= 46) | | *p* value |
| --- | --- | --- | --- | --- | --- |
|  | n | (%) | n | (%) |  |
| Age |  |  |  |  |  |
| Median | 52 |  | 50 |  | 0.16 |
| Range | (31-74) |  | (24-77) |  |  |
| ECOG performance |  |  |  |  |  |
| 0 | 31 | 70.5 | 24 | 52.2 | 0.13 |
| 1 | 12 | 27.3 | 21 | 45.7 |  |
| 2 | 1 | 2.2 | 1 | 2.2 |  |
| Pathologic findings |  |  |  |  |  |
| Squamous cell carcinoma (SCC) | 41 | 93.2 | 43 | 93.5 | 1 |
| Large cell keratinizing | 9 | 20.5 | 9 | 19.6 |  |
| Large cell non-keratinizing | 26 | 59.1 | 21 | 45.7 |  |
| Large cell, not specified | 0 | 0 | 3 | 6.5 |  |
| SCC, not specified | 6 | 13.6 | 8 | 17.4 |  |
| Small cell | 0 | 0 | 2 | 4.3 |  |
| Others (adenocarcinoma, adenosquamous carcinoma) | 3 | 6.8 | 3 | 6.5 |  |
| Tumor shape |  |  |  |  |  |
| Exophytic | 22 | 50 | 16 | 34.8 | 0.14 |
| Infiltrative | 22 | 50 | 30 | 65.2 |  |
| Parametrial involvement |  |  |  |  |  |
| No | 7 | 15.9 | 3 | 6.5 | 0.19 |
| Yes | 37 | 84.1 | 43 | 93.5 |  |
| Unilateral | 21 | 47.7 | 21 | 45.7 |  |
| Bilateral | 16 | 36.4 | 22 | 47.8 |  |
| Endocervical extension |  |  |  |  |  |
| No | 2 | 4.5 | 4 | 8.7 | 0.45 |
| Yes | 16 | 36.4 | 21 | 45.7 |  |
| Not confirmed | 26 | 59.1 | 21 | 45.7 |  |
| Primary tumor size (cm) |  |  |  |  |  |
| Median | 5 |  | 5 |  | 0.61 |
| Range | (2-10) |  | (2-9) |  |  |
| Pelvic LN involvement | 30 | 68.2 | 38 | 82.6 | 0.11 |
| Paraaortic LN involvement | 44 | 100 | 46 | 100 | 1 |
| Hydronephrosis | 12 | 27.3 | 6 | 13 | 0.09 |
| FIGO stage |  |  |  |  |  |
| IB | 7 | 15.9 | 3 | 6.5 | <0.001 |
| IIB | 9 | 20.5 | 30 | 65.2 |  |
| IIIA | 1 | 2.2 | 1 | 2.2 |  |
| IIIB | 22 | 50 | 11 | 23.9 |  |
| IVA | 5 | 11.4 | 1 | 2.2 |  |
| Treatment period |  |  |  |  |  |
| Before 2000 | 40 | 90.9 | 12 | 26.1 | <0.001 |
| 2000-present | 4 | 9.1 | 34 | 73.9 |  |
| Total dose to Point A (Gy) |  |  |  |  |  |
| Median | 69.6 |  | 72 |  | 0.98 |
| Range | (56-94.2) |  | (56-93) |  |  |
| Total dose to para-aortic lymphatics |  |  |  |  |  |
| Median | 54 |  | 50.4 |  | 0.62 |
| Range | (45-60.4) |  | (45-60) |  |  |

Abbreviation: ECOG, Eastern Cooperative Oncology Group performance; EFRT, extended-field radiation therapy; CTx, concurrent chemotherapy

Table S3 Salvage treatments after first recurrence according to treatment modality in relapsed patients

| Variables | EFRT alone (n=21) | | EFRT withs CTx (n=25) | | *p* value |
| --- | --- | --- | --- | --- | --- |
|  | n | (%) | n | (%) |  |
| All salvage treatments | 11 | 52.4 | 20 | 80.0 | 0.047 |
| Salvage chemotherapy | 5 | 23.8 | 16 | 64 | 0.006 |
| Salvage radiotherapy | 7 | 33.3 | 14 | 56 | 0.12 |
| Tumor removal | 2 | 9.5 | 2 | 8 | 1 |
| Only conservative care | 8 | 38.1 | 5 | 20 | 0.18 |

Abbreviation: EFRT, extended-field radiation therapy; CTx, concurrent chemotherapy
