# Supplementary figures and images for: Treatment outcomes of extended-field radiation therapy and the effect of concurrent chemotherapy on uterine cervical cancer with para-aortic lymph node metastasis
Source: Radiat Oncol. 2015 Jan 13;10:18. doi: 10.1186/s13014-014-0320-5 (PMC4311470; doi:10.1186/s13014-014-0320-5)

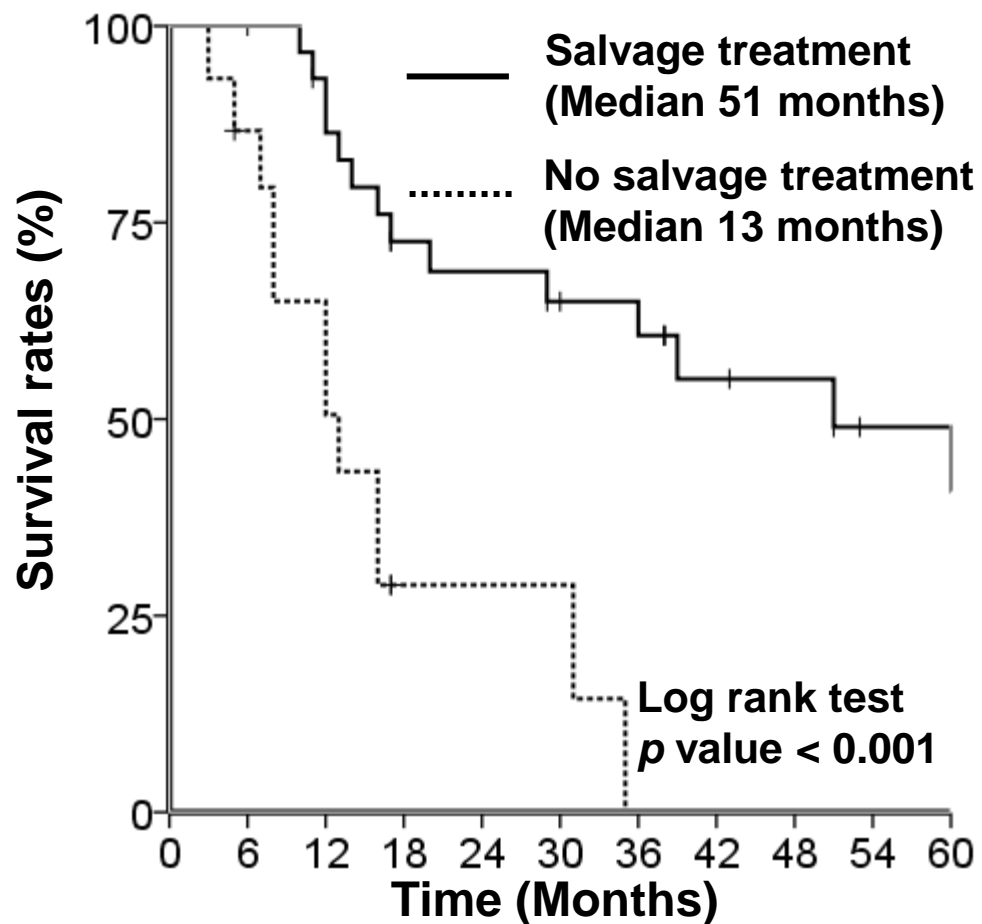

#### No. at Risk

|                      |    |    |    |    |    |    |    |    |   |   |   |
|----------------------|----|----|----|----|----|----|----|----|---|---|---|
| Salvage treatment    | 31 | 30 | 26 | 19 | 18 | 16 | 14 | 10 | 8 | 6 | 4 |
| No salvage treatment | 15 | 12 | 8  | 2  | 2  | 2  | 0  | 0  | 0 | 0 | 0 |

Supplement: Additional file 2: Figure S1. — Kaplan–Meier curve depicts that patients receiving salvage treatment showed significantly improved overall survival rates compared to those receiving only conservative care after relapse (median OS 51 vs. 13 months, p < 0.001). [file 13014_2014_320_MOESM2_ESM.pdf]
